# Multidimensional Alternating Kernel Method for cortical layer segmentation in 3D reconstructed histology

**DOI:** 10.1016/j.mex.2024.102674

**Published:** 2024-04-05

**Authors:** Kwame S. Kutten, Jenny Trieu, Jaden Dawson, Lisa Hou, Lea Sollmann, Andrej Kral, Peter Hubka, J. Tilak Ratnanather

**Affiliations:** a Johns Hopkins University, Baltimore, MD, USA; b Morgan State University, Baltimore, MD, USA; c Hannover Medical School, Hannover, Germany

**Keywords:** Alternating Kernel Method, Auditory cortex, Hearing loss, Histology, Segmentation, Multidimensional Alternating Kernel Method

## Abstract

The neocortex of the brain can be divided into six layers each with a distinct cell composition and connectivity pattern. Recently, sensory deprivation, including congenital deafness, has been shown to alter cortical structure (e.g. the cortical thickness) of the feline auditory cortex with variable and inconsistent results. Thus, understanding these complex changes will require further study of the constituent cortical layers in three-dimensional space. Further progress crucially depends on the use of objective computational techniques that can reliably characterize spatial properties of the complex cortical structure. Here a method for cortical laminar segmentation is derived and applied to the three-dimensional cortical areas reconstructed from a series of histological sections from four feline brains. In this approach, the Alternating Kernel Method was extended to fit a multi-variate Gaussian mixture model to a feature space consisting of both staining intensity and a biologically plausible equivolumetric depth map.

This research method

• Extends the Alternating Kernel Method to multi-dimensional feature spaces.
• Uses it to segment the cortical layers in reconstructed histology volume. Segmentation features include staining intensity and a biologically plausible equivolumetric depth map.
• Validates results in auditory cortical areas of feline brains, two with normal hearing and two with congenital deafness.

## Graphical abstract

**Figure:**
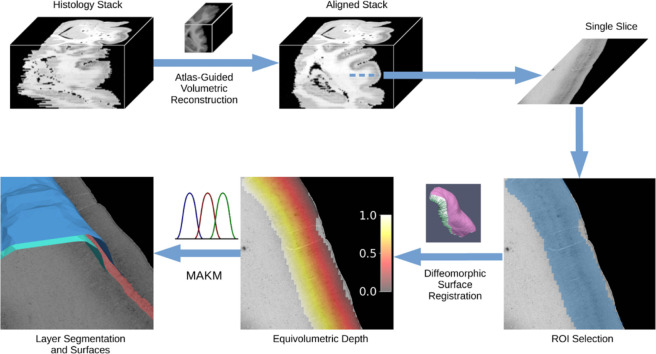

Specifications table

**Table:** 

Subject area:	Bioinformatics
More specific subject area:	Image Segmentation
Name of your method:	Multidimensional Alternating Kernel Method
Name and reference of original method:	Name: Alternating Kernel Method Reference: C. E. Priebe and D. J. Marchette, “Alternating kernel and mixture density estimates,” Comput Stat Data Anal, vol. 35, no. 1, pp. 43–65, 2000, doi: 10.1016/S0167–9473(00)00003–7. Link: https://doi.org/10.1016/S0167–9473(00)00003–7
Resource availability:	Data and code available upon request

## Method details

### Background

**Fig. 1:**
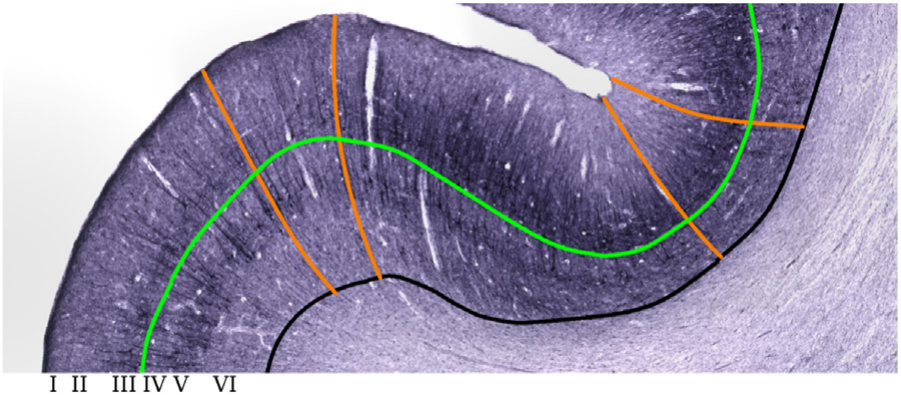
Schematic of curved laminar coordinate system overlaid on SMI-32 histological sections. Roman numerals identify layer locations. Orange lines denote curved paths along cortical columns. Black and green lines denote gray-white and layer III-IV boundaries respectively. (For interpretation of the references to color in this figure legend, the reader is referred to the web version of this article.)

Much of the cerebral cortex, called neocortex, can be divided into six layers traditionally identified by Roman numerals with increasing depth from I to VI (Fig. 1). Laminar structure and thickness are closely related to information processing in the cerebral cortex, which underlies many cognitive processes including sensory perception and behavior [1,2]. However, an exact link between the cortical layer structure and cortical function is not settled yet. For example, differences in the hearing experience are known to alter activation patterns in the auditory cortex [3,4]. Prior histological studies have shown that congenital deafness alters auditory cortex laminar thickness with inconsistent results [5], [6], [7], [8], [9]. Thus, finding a reliable method for cortical layer segmentation could potentially resolve this problem.

It was recently shown that cortical layers III, V and VI mostly drives cortical thicknesses diversity in sensory, motor and association areas [10,11]. This was done through whole-brain laminar segmentation of a silver-stained human brain (BigBrain) histological atlas via a convolutional neural network trained on 51 manually segmented regions. A different approach took advantage of the rich amount of information available in diffusion MRI [12]. Layers were separated through k-means clustering based on 31 diffusivity and orientation parameters. Classification did not use spatial or depth information but instead relied on the high-dimensional parameter profiles being specific for different cortical layers. Here we propose a segmentation approach based on the observation that layers often cannot be distinguished using intensity alone in many histological stains. For example, layers III and V are both darkly stained by SMI-32 due to the presence of pyramidal cell soma [13] and consequently cannot be reliably distinguished using staining intensity alone (Fig. 1). Hence combining staining information with a biologically and geometrically consistent depth map (i.e. depth profile) from readily available tools may provide a convenient way to create laminar segmentation from histological data.

### Image acquisition

**Fig. 2:**
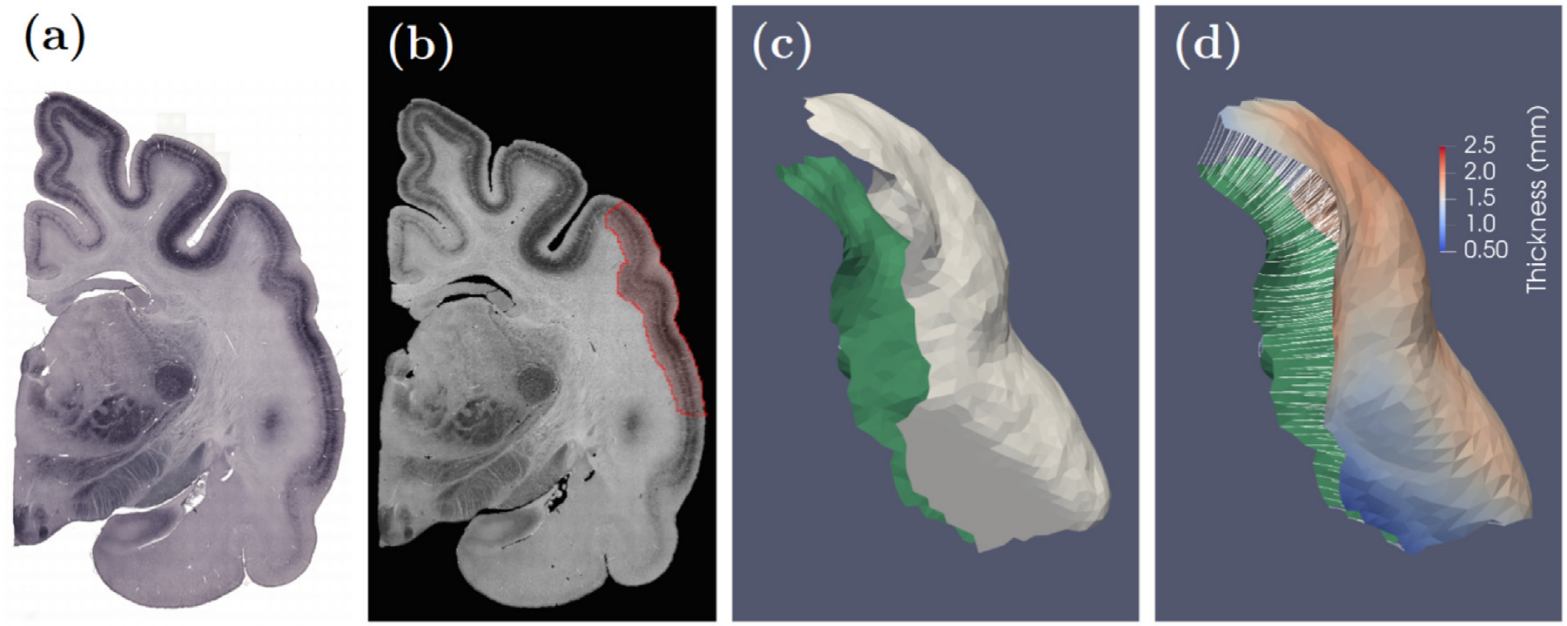
(a) Original SMI Slice from a feline brain; (b) Region of interest consisting of DZ and A1 and A2 auditory cortical areas (marked in red); (c) Inner and outer cortical surfaces; (d) Cortical thickness (shown on the outer surface) are the lengths of the normal curves (shown in white) generated by constrained diffeomorphic evolution of the inner surface towards the outer one. (For interpretation of the references to color in this figure legend, the reader is referred to the web version of this article.)

Feline experiments were approved by the local state authorities and were performed in compliance with the guidelines of the European Community European Union legislation for the care and use of laboratory animals (EU Directive 2010/63/EU), ARRIVE guidelines and the German Animal Welfare Act (TierSchG). Coronal sections of feline brain (right hemispheres) comprising all auditory cortical areas were collected. SMI-32 staining was used to identify cortical pyramidal cells [14] (Fig. 2a). Sixty SMI-32 stained slices, each 50 µm thick were obtained at 250 µm intervals. Overlapping image tiles were acquired under light microscopy at ∼1 µm isotropic resolution. They were then merged into whole slices using TeraStitcher [15].

### Volumetric reconstruction

**Fig. 3:**
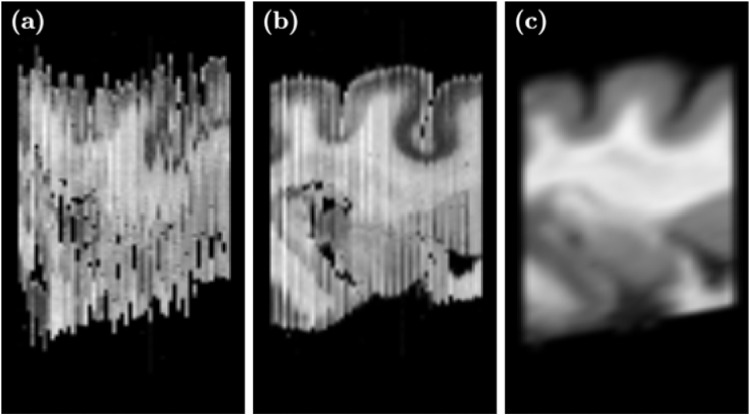
Volumetric reconstruction visualized in axial direction. (a) Histology before slice alignment, (b) histology after shape-guided alignment, and (c) cropped MR atlas used as shape prior.

The volumetric reconstruction of a curved object from two-dimensional slices is ambiguous without a shape prior [16]. Thus, this was done with the help of a volumetric feline brain atlas [17] consisting of a T1-weighted MR volume with 71 labeled brain regions. Histological images from four cats, two with normal hearing and two with congenital hearing loss, were volumetrically reconstructed in the following way. For each brain, the atlas was cropped to the brain region covered by the histology. Next the atlas was registered to the grayscale histology under a mean square error metric. Rigid histological slices were then aligned under a cost function, which simultaneously considered the error between adjacent slices and the MR volume [18] (Fig. 3).

A region of interest consisting of the dorsal zone (DZ), primary (A1) and secondary auditory cortices (A2) was then isolated using the atlas labels followed by manual correction (Fig. 2b). This region was converted into a closed surface using restricted Delaunay triangulation [19]. This surface was cut along geodesic paths [20] into inner and outer surfaces at the gray-white and pial cortical surface respectively (Fig. 2c).

### Equivolumetric depth

It has been hypothesized that cortical layer morphology varies due to equivolumetric deformation within cortical columns [21,22]. In gyral columns, a constant volume ratio is maintained because outer regions are wider due to layers being thinner while inner regions are narrower due to layers being thicker. The opposite effect is observed in sulcal columns (Fig. 1). Under this assumption, several methods for computing equivolumetric depth from images have been developed. These techniques can be broadly divided into two categories: voxel-based or surface-based. In voxel-based approaches, equivolumetric depth is approximated directly within the voxels of the image [23], [24], [25] while surface-based methods operate on triangulated meshes reconstructed from image data [26], [27], [28]. Here, we use a surface-based approach in which diffeomorphic registration of inner surfaces to pial surfaces was applied to create one-to-one non-intersecting trajectories within the cortex [29]. These trajectories were defined on a time-domain t∈[0,1] from inner to outer surfaces with normality constraints enforced at each end. Equivolumetric depth was obtained through reparameterization of intermediate deformed surfaces to maintain constant volume along trajectories [30]. A corresponding depth image with range from 0 at the pia surface to 1 at gray-white matter boundary was then created by interpolating registration trajectories on a regular grid.

### Joint histogram

**Fig. 4:**
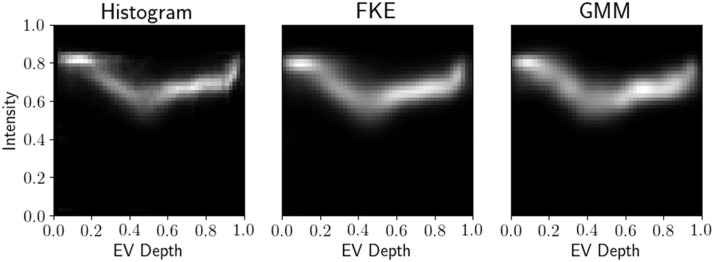
Joint histogram q of grayscale intensity and equivolumetric depth, followed by corresponding FKE $f_{{\hat{\theta }}_{M}}$ and GMM $g_{{\hat{\theta }}_{M}}$ after optimization with M=12 components.

The equivolumetric depth and grayscale histology were combined into a multi-channel image. In the general case we let $I:\Omega \to Y\subset R^{N}$ represent a multi-channel image such that $I=[I_{1},\cdots ,I_{N}]$*.* Each d-dimensional image channel has the same domain $\Omega \subset R^{d}$ such that $I_{n}:\Omega \to R$. In this specific work N=2 with equivolumetric depth as $I_{1}$ and grayscale histology $I_{2}$. The joint histogram of I, q:Y→[0,∞) is defined as a probability density such that $\int_{y\in Y}q\left(y\right)\mathrm{dy}=1$. Intuitively this histogram q can be understood as the probability voxels in I have intensity value of y. An example joint histogram computed using 50 evenly spaced bins is depicted in the first plot of Fig. 4.

Our goal is to approximate the parameters $\theta_{M}$ of an M-component Gaussian Mixture Model (GMM) $g_{\theta_{M}}:Y\to R$ that could have generated the joint histogram. It is defined as

(1) $$g_{\theta_{m}}=\sum_{m=1}^{M}\omega_{m}\psi_{K_{m}}\left(y-\mu_{m}\right)$$

where $\psi_{K_{m}}:Y\to R$ is the m^th^ zero-centered multivariate Gaussian probability density function with covariance $K_{m}\in R^{N\times N}$. Furthermore $\mu_{m}\in R^{N}$ are the means and $\omega_{m}\in \left[0,1\right]$ are the weights such that $\sum_{m=2}^{M}\omega_{m}=1$. Thus, our full set of parameters is defined as $\theta_{m}=\left\{\omega_{1},\cdots ,\omega_{M},\mu_{1},\cdots ,\;\mu_{M},K_{1},\cdots ,K_{m}\right\}$.

### Algorithm

Given constant bandwidth, denoted by diagonal matrix $h=\text{diag}(h_{1},\ldots ,h_{N})$, the Filtered Kernel Estimate (FKE) is defined as

(2) $$f_{\theta_{M}}\left(y\right)=\sum_{m=1}^{M}\left(\left(\rho_{m}q\right)*\psi_{h}K_{m}\right)\left(y\right)$$

where $\rho_{m}\left(y\right)=\omega_{m}\psi_{K_{m}}\left(y-\mu_{m}\right)g_{\theta_{M}}^{-1}\left(y\right)$ are filter functions and * denotes the convolution. The Alternating Kernel Method (AKM) adds components one at a time estimating new parameters ${\hat{\theta }}_{M}$ which minimize the distance between the current GMM (1) and previous FKE (2). Thus, starting at M=1 we find subsequent components by

(3) $${\hat{\theta }}_{M}=\underset{\theta_{m}}{\text{argmin}}{∥f_{{\hat{\theta }}_{M-1}}-g_{\theta_{M}}∥}_{L_{2}}^{2}$$

using gradient descent assuming that $f_{{\hat{\theta }}_{0}}=q$ (more details found in supplementary material). This process continues until the final number of components $M=M_{\text{max}}$ is attained. An example GMM and corresponding FKE after fitting to a joint histogram is depicted in Fig. 4.

### Initialization

Gradient descent optimization of Eq. (3) starts at an initial guess of parameters $\theta_{M}=\left\{w_{M},\;\mu_{M},\;K_{M}\right\}\cup \left\{{\hat{\theta }}_{M-1}\right\}$. These initial values were found automatically by modifying the “smart start” approach described previously [31]. First a new component with center $\mu_{M}$ is added where the error $e=f_{{\hat{\theta }}_{M-1}}-g_{\theta_{M-1}}$is greatest. In the original implementation, the histogram domain Y was one-dimensional and could be trivially divided into disjoint regions in which e>0. A new component was then inserted in the region of maximum error. Here in our multidimensional setting, Y has a more complex topology and thus a different approach was needed. Let b:Y→R be a centered-box kernel with unit sum. The convolution (e*b)(y) can be interpreted as the average error within a region centered on y. Thus, the initial guess for the center of the new component is $\mu_{M}={\text{argmax}}_{y\in Y}\left(e*b\right)\left(y\right)$.

Once an initial $\mu_{M}$ has been found, we assign the component a weight of $w_{m}=\left(e*b\right)\left(\mu_{M}\right)$ to ensure that the error is canceled out. Elements of covariance matrix $K_{m}$ are then initialized to ${\left(K_{m}\right)}_{ij}=w_{M}^{-1}\sum_{y\in Y}e\left(y\right)b\left(y-\mu_{M}\right)\left(y_{i}-{\left(\mu_{M}\right)}_{i}\right)\left(y_{j}-{\left(\mu_{M}\right)}_{j}\right)$ where ${\left(\mu_{M}\right)}_{i}\in R$ and ${\left(\mu_{M}\right)}_{j}\in R$ are the i^th^ and j^th^ elements of mean vector $\mu_{M}$ respectively.

### Segmentation

After convergence, a segmentation label image $L:\;\Omega \;\to \;\{1,\;.\;.\;.,\;M_{\text{max}}\}$ can be generated for multi-channel image I. In the general case this can be done by

$$L\left(x\right)=\underset{m\in \left\{1,\;.\;.\;.,\;M_{\max}\right\}\;}{\text{argmax}}\rho_{m}\left(I\left(x\right)\right)$$

But in specific case of laminar segmentation, this formulation can lead to unnatural results as subsequent labels should be contiguous (e.g. label 4 should always occur between labels 3 and 5). Suppose our components have been sorted by increasing depth from m=1 to $M_{\text{max}}$. Furthermore, suppose for a specific voxel $x_{0}\in \Omega$ we have $\rho_{3}\left(I(x_{0})\right)=0.51$, $\rho_{5}\left(I(x_{0})\right)=0.49$ and $\rho_{m}\left(I(x_{0})\right)=0$ for m∉{3,5}. Ideally a label of 4 should be assigned to this voxel since it is clearly between components 3 and 5. But instead the above equation would give us $L(x_{0})=3$. Thus, we adopt

(4) $$L\left(x\right)=\left\lfloor \sum_{m=1}^{M_{\text{max}}}m\rho_{m}\left(I\left(x\right)\right)\right\rceil$$

where the ⌊·⌉ operation rounds values to the nearest integer. In the aforementioned example this would give us the desired result of $L(x_{0})=4$.

### Method validation

**Fig. 5:**
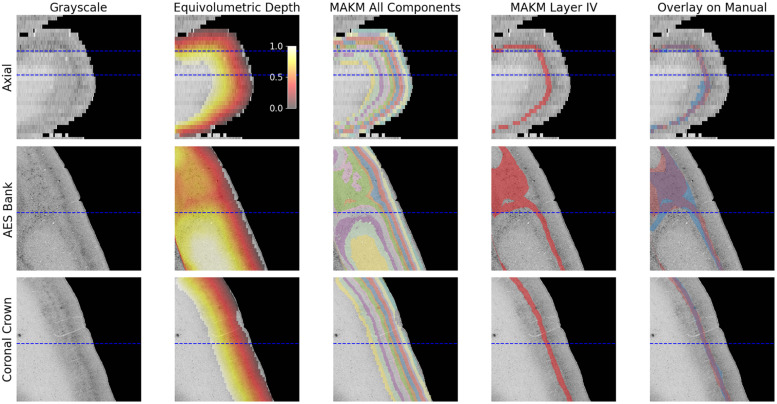
Results for a cat with hearing loss. Rows are axial slice, coronal slice through AES bank and coronal slice through gyral crown respectively. Columns are grayscale image, equivolumetric depth, MAKM 12-component segmentation and overlay on manual segmentation. Dashed lines indicate where axial and coronal slices intersect.

Grayscale histology from one of the deaf cats is shown in Fig. 5. The first column depicts an axial slice through the region of interest followed by a coronal slice through the anterior ectosylvian sucal (AES) bank and then gyral crown. The second column shows the corresponding overlay of equivolumetric depth. The resulting multi-component segmentation is shown in the third column of (Fig. 5). The 8th and 9th components clearly represented layer IV as shown in the fourth column, while an overlay of the manual segmentation is found in the fifth column.

Dice overlap [32] between manual and AKM segmentations were computed for all four brains. Average and standard deviation in these values across all four cats was 0.54±0.03. Higher agreement 0.61±0.03 was observed in gyral crown regions when compared to 0.50±0.04 and 0.45±0.10 found in the banks of the anterior (AES) and posterior ectosylvian sulci (PES) respectively (Supplementary Table 1). Dice scores of <0.7 were likely caused by well-known limitations of the Dice metric for smaller regions of interest [33]. Additionally, in sulcal banks, partial volume effects made segmentation more difficult due to lower resolution in slicing direction. Coronal slice planes were 0.25 mm apart while layer IV was typically 0.2 - 0.3 mm thick.

**Supplementary Fig. 6:**
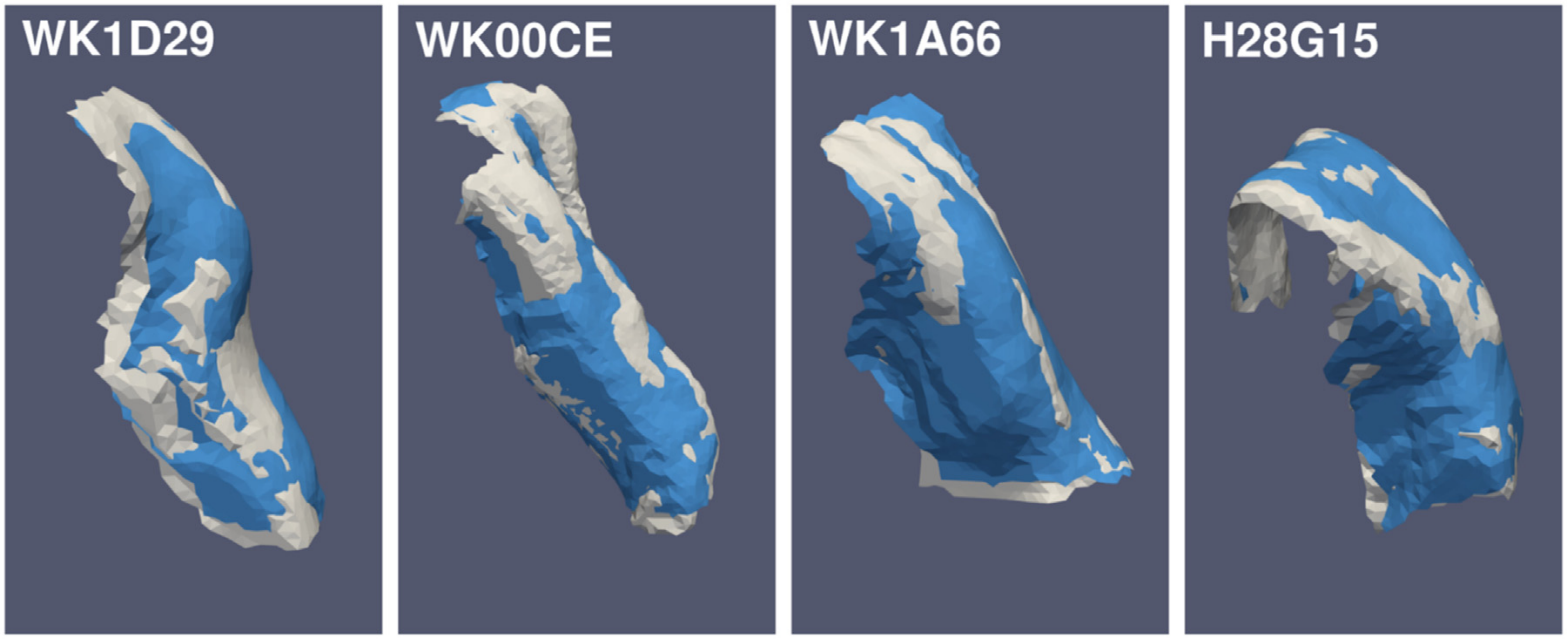
Comparison of layer III/IV interface surfaces from manual (white) and AKM (blue) segmentations. (For interpretation of the references to color in this figure legend, the reader is referred to the web version of this article.)

**Supplementary Fig. 7:**
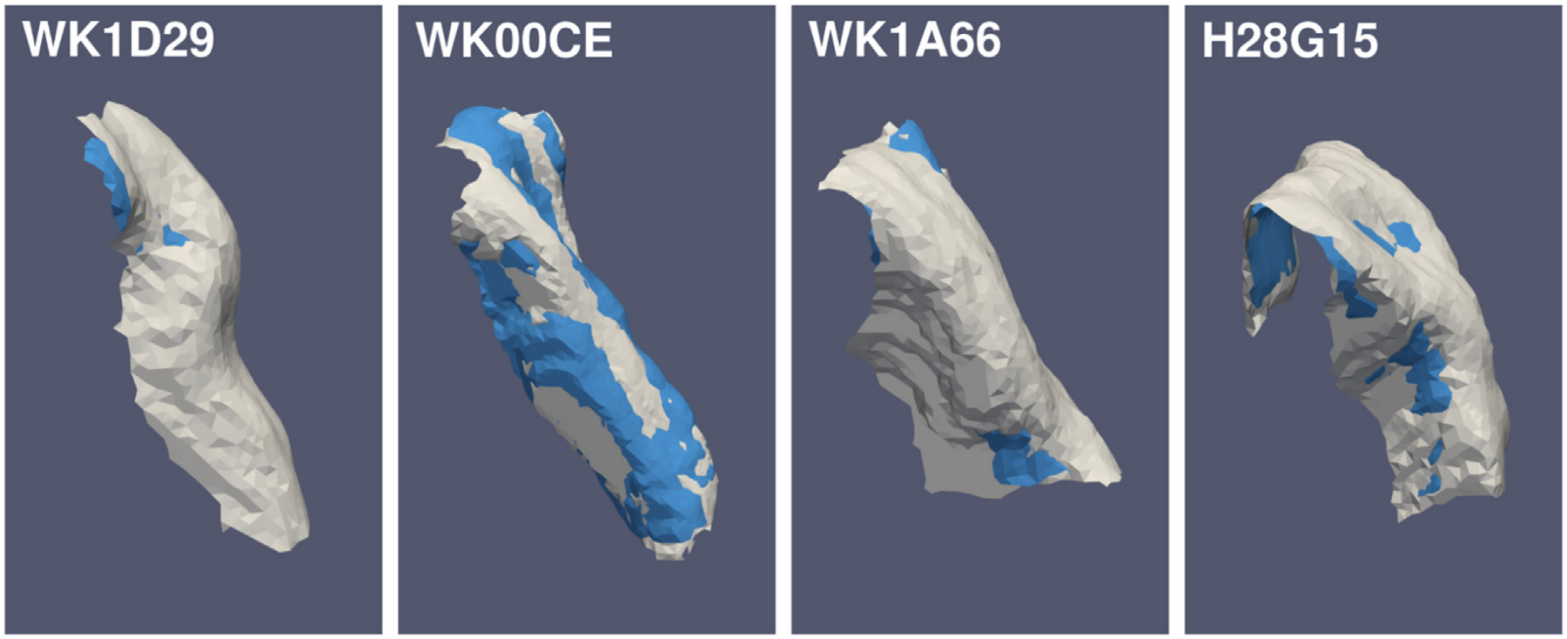
Comparison of layer IV/V interface surfaces from manual (white) and AKM (blue) segmentations. (For interpretation of the references to color in this figure legend, the reader is referred to the web version of this article.)

Layer III/IV and IV/V interface surfaces were generated using restricted Delaunay triangulation for both the manual and AKM segmentations (Supplementary Figs. 6 and 7). They were then compared using the Hausdorff distance. Since this metric is known for being sensitive to outliers [34], particularly along the edge of the surfaces, the metric at 50th, 75th and 95th distance percentiles were calculated in additional to the traditional 100th percentile for the maximum distance. Across all brains, the mean and standard deviation for the 50th and 75th percentiles were 0.204±0.043 mm and 0.315±0.092 mm respectively (Supplementary Table 2) as compared to a slice separation of 0.25 mm which suggests that layer IV can be reliably reconstructed in 3D.

A limitation of our segmentation approach is the reliance on an adequate spatial model in the form of an equivolumetric depth map. While in laminar segmentation a spatial model can be defined based on equivolumetric theory, this is not feasible in other biomedical segmentation problems. Furthermore, the class of kernel, Gaussian in our approach, is assumed a priori and limits the number of features that could be evaluated. Therefore, generalizing our method would require much more sophisticated methods with generalized kernels such as a U-Net trained on multichannel input images [35]. Qualitative agreement of algorithmic and manual segmentations demonstrates the potential for this technique to separating supragranular layers I-III from infragranular layers V-VI. Its utility in isolating individual cortical layers is more limited due to lower metric values.

## CRediT authorship contribution statement

**Kwame S. Kutten:** Conceptualization, Methodology, Software, Visualization, Writing – original draft. **Jenny Trieu:** Software, Data curation. **Jaden Dawson:** Data curation. **Lisa Hou:** Data curation. **Lea Sollmann:** Resources, Data curation. **Andrej Kral:** Supervision, Writing – review & editing, Funding acquisition. **Peter Hubka:** Conceptualization, Supervision, Funding acquisition, Resources, Data curation, Writing – review & editing. **J. Tilak Ratnanather:** Conceptualization, Methodology, Software, Visualization, Supervision, Writing – review & editing, Funding acquisition.

## Declaration of competing interest

The authors declare that they have no known competing financial interests or personal relationships that could have appeared to influence the work reported in this paper.

## Figures and Tables

**Supplementary Table 1:** Dice scores for the entire for the entire (overall) region, gyral crown, AES bank and PES bank.

Brain ID	Sex	Hearing	Dice overlap			
			Overall	Crown	AES Bank	PES Bank
WK1D29	Female	No	0.54	0.56	0.51	0.55
WK00CE	Female	No	0.56	0.62	0.55	0.53
WK1A66	Male	Yes	0.48	0.64	0.46	0.31
H28G15	Female	Yes	0.56	0.63	0.46	0.41
Mean±SD			0.54±0.03	0.61±0.03	0.50±0.04	0.45±0.10

**Supplementary Table 2:** Interface surface Hausdorff distances in mm calculated at given percentile of distances.

Brain ID	Surface	Hausdorff distance (mm) at given percentile			
		50 %	75 %	95 %	100 %
WK1D29	III/IV	0.168	0.248	0.445	1.277
	IV/V	0.177	0.261	0.749	1.864
WK00CE	III/IV	0.184	0.27	0.514	1.21
	IV/V	0.278	0.537	1.623	2.476
WK1A66	III/IV	0.164	0.259	0.522	1.176
	IV/V	0.273	0.372	0.733	1.12
H28G15	III/IV	0.191	0.301	0.684	1.576
	IV/V	0.195	0.273	0.543	1.458
Mean±SD		0.204±0.043	0.315±0.092	0.727±0.355	1.520±0.429

## Data Availability

Data will be made available on request.

## Supplementary material *and/or* additional information

### Gradient descent

Starting from $\theta_{M}=\left\{w_{M},\;\mu_{M},\;K_{M}\right\}\cup \left\{{\hat{\theta }}_{M-1}\right\}$, we minimize Eq. (3) through gradient descent in terms of all parameters in $\theta_{M}$. Given zero-centered multivariate Gaussian probability density $\psi_{K_{m}}:Y\to \;R$ is

$$\psi_{K_{m}}\left(y\right)={\left(2\pi \right)}^{-N/2}{\left|K_{m}\right|}^{-1/2}\exp\left(-\frac{1}{2}\;y^{T}K_{m}^{-1}y\right)$$

where $|K_{m}|$ is the determinant of the covariant matrix. The gradient for the m^th^ component weight is

$$\Delta w_{m}=\frac{\partial }{\partial w_{m}}{∥f_{{\hat{\theta }}_{M-1}}-g_{\theta_{M}}∥}_{L_{2}}^{2}=2\sum_{y\in Y}\left(f_{{\hat{\theta }}_{M-1}}\left(y\right)-g_{\theta_{M}}\left(y\right)\right)\psi_{K_{m}}\left(y-\mu_{m}\right)$$

By the chain rule we can find gradient for mean $\mu_{m}\in R^{N}$

$$\Delta \mu_{m}=\frac{\partial }{\partial w_{m}}{∥f_{{\hat{\theta }}_{M-1}}-g_{\theta_{M}}∥}_{L_{2}}^{2}=2w_{m}\sum_{y\in Y}\left(f_{{\hat{\theta }}_{M-1}}\left(y\right)-g_{\theta_{M}}\left(y\right)\right)\psi_{K_{m}}\left(y-\mu_{m}\right)K_{m}^{-1}\left(y-\mu_{m}\right)$$

For $K_{m}\in \;R^{N\times N}$ we differentiate taking advantage of the matrix identities $\frac{d}{dK_{m}}|K_{m}\left|=\right|K_{m}|{\left(K_{m}^{-1}\right)}^{T}$ and $\frac{d}{dK_{m}}\left(y^{T}K_{m}^{-1}y\right)=-{\left(K_{m}^{-1}\right)}^{T}yy^{T}{\left(K_{m}^{-1}\right)}^{T}$ [36] with the symmetry of the covariance matrix guaranteeing ${\left(K_{m}^{-1}\right)}^{T}=K_{m}^{-1}$. Thus, the gradient is

$$\Delta K_{m}=\frac{\partial }{\partial w_{m}}{∥f_{{\hat{\theta }}_{M-1}}-g_{\theta_{M}}∥}_{L_{2}}^{2}=w_{m}\sum_{y\in Y}\left(f_{{\hat{\theta }}_{M-1}}\left(y\right)-g_{\theta_{M}}\left(y\right)\right)\psi_{K_{m}}\left(y-\mu_{m}\right)\left(K_{m}^{-1}\left(y-\mu_{m}\right){\left(y-\mu_{m}\right)}^{T}K_{m}^{-1}-K_{m}^{-1}\right)\;$$

Therefore, we can find updated values of each parameter $\left(w_{m}^{*},\mu_{m}^{*},K_{m}^{*}\right)=\left(w_{m}+\epsilon_{w}\Delta w_{m},\;\mu_{m}+\epsilon_{\mu }\Delta \mu_{m},\;K_{m}+\epsilon_{K}\Delta K_{m}\right)$ where $\epsilon_{w},\;\epsilon_{\mu },\;\epsilon_{K}\in \left(0,\infty \right)$ are learning rates for the weights, means and covariances respectively. If $∥f_{{\hat{\theta }}_{M-1}}-g_{\theta_{M}^{*}}{∥}_{L_{2}}<∥f_{{\hat{\theta }}_{M-1}}-g_{\theta_{M}}{∥}_{L_{2}}$ we take $\theta_{M}=\theta_{M}^{*}$ and advance to the next iteration and slightly increase the learning rates. Otherwise, we cut the learning rates in half and attempt the update again. This process continues until the maximum number of iterations is exceeded or the learning rate falls below some predefined threshold.

### Comparison metrics

Manual and AKM segmentations and their corresponding reconstructed surfaces were compared using Dice overlap and Hausdorff distances respectively. Supplementary Table 1 shows Dice overlap for four brains within specified regions. Surfaces for Layer III/IV (Supplementary Fig. 6) and Layer IV/V (Supplementary Fig. 7) were generated from manual and AKM segmentations and Supplementary Table 2 displays Haudsorff distances between these surfaces in mm calculated given distance percentiles. Note that the 100 % percentile is calculated at the maximum and represents the traditional Hausdorff metric.
